# Remifentanil up‐regulates HIF1α expression to ameliorate hepatic ischaemia/reperfusion injury via the ZEB1/LIF axis

**DOI:** 10.1111/jcmm.15929

**Published:** 2020-09-30

**Authors:** Rongsheng Zhou, Shuang Li, Xiaopeng Mei, Tao Jiang, Qiang Wang

**Affiliations:** ^1^ Department of Anesthesiology The First Affiliated Hospital of Xi’an Jiaotong University Xi’an China

**Keywords:** hepatic ischaemia/reperfusion injury, HIF1α, LIF, remifentanil, ZEB1

## Abstract

Ischaemia/reperfusion (I/R)‐induced hepatic injury is regarded as a main reason of hepatic failure after transplantation or lobectomy. The current study aimed to investigate how the opioid analgesic remifentanil treatment affects I/R‐induced hepatic injury and explore the possible mechanisms related to HIF1α. Initially, an I/R‐induced hepatic injury animal model was established in C57BL/6 mice, and an in vitro hypoxia‐reoxygenation model was constructed in NCTC‐1469 cells, followed by remifentanil treatment and HIF1α silencing treatment. The levels of blood glucose, lipids, alanine transaminase (ALT) and aspartate transaminase (AST) in mouse serum were measured using automatic chemistry analyser, while the viability and apoptosis of cells were detected using CCK8 assay and flow cytometry. Our results revealed that mice with I/R‐induced hepatic injury showed higher serum levels of blood glucose, lipids, ALT and AST and leukaemia inhibitory factor (LIF) expression, and lower HIF1α and ZEB1 expression (*P* < .05), which were reversed after remifentanil treatment (*P* < .05). Besides, HIF1α silencing increased the serum levels of blood glucose, lipids, ALT and AST (*P* < .05). Furthermore, hypoxia‐induced NCTC‐1469 cells exhibited decreased HIF1α and ZEB1 expression, reduced cell viability, as well as increased LIF expression and cell apoptosis (*P* < .05), which were reversed by remifentanil treatment (*P* < .05). Moreover, HIF1α silencing down‐regulated ZEB1 expression, decreased cell viability, and increased cell apoptosis (*P* < .05). ZEB1 was identified to bind to the promoter region of LIF and inhibit its expression. In summary, remifentanil protects against hepatic I/R injury through HIF1α and downstream effectors.

## 1 INTRODUCTION

Ischaemia/reperfusion (I/R)‐induced hepatic injury is a major cause of hepatic failure after transplantation or lobectomy.^1^, ^2^ Ischaemia due to interrupted blood supply to the liver results in rapid dysfunction of oxygen‐dependent hepatocytes, but subsequent reperfusion ultimately promotes inflammation and cell death.^3^, ^4^ I/R injury of the organ could result from many factors, for example the release of free oxygen radicals and lipid peroxidation, cell death, inflammatory mediators and microvasculature damage.^5^ In recent years, peroxisome proliferator activated receptor gamma (PPARγ), long noncoding RNAs (lncRNAs), and microRNAs (miRNAs) have been reported to play important roles to relieve hepatic I/R injury.^6^, ^7^, ^8^ In animal experiments, ischaemic pre‐conditioning and low‐temperature reperfusion alleviated hepatic I/R injury, but these preclinical findings are not yet translatable to clinical procedures.^9^, ^10^ Currently, there are few effective therapeutic strategies for protecting against I/R‐induced hepatic injury.^2^

Remifentanil (Ultiva™) is a selective μ‐opioid receptor agonist, which is used clinically for analgesia and as part of general anaesthesia.^11^, ^12^, ^13^ Remifentanil reportedly protects the heart from I/R injury via post‐conditioning^14^ and reduces hepatic I/R injury in rats.^15^ However, the molecular mechanism by which remifentanil attenuates hepatic I/R injury is not fully understood. Daijo et al^16^ showed that the expression of hypoxia‐inducible factor 1α (HIF1α) was up‐regulated by remifentanil. Guo et al^17^ summarized current studies regarding the role of HIF1α in hepatic I/R injury, which indicated that stabilization of HIF1α can attenuate hepatic I/R injury. HIF1α belongs to the HIF transcription factor family, which participates in a variety of biological processes such as cell survival under hypoxic condition, glycolysis and angiogenesis.^18^, ^19^, ^20^ Under hypoxia, HIF1α forms a heterodimer with HIF1β, which binds to hypoxia‐responsive elements (HREs) in the promoter regions of downstream target genes.^21^ In cortical ischaemia, up‐regulation of Zinc finger E‐box binding homeobox 1 (ZEB1) mRNA and protein was shown to be a protective response to ischaemia by neurons.^22^ In glioblastoma, HIF1α could induce the expression of ZEB1, which is a member of zinc finger homeodomain transcription factor family and plays important roles in the epithelial‐mesenchymal transition (EMT) of embryogenesis.^23^ ZEB1 was also found to regulate EMT in epithelial cancers.^24^ Edward et al^25^ reported that ZEB1 binding sites were located within the leukaemia inhibitory factor (LIF) promoter region and could inhibit LIF expression in glioma cancer stem cells. LIF was first described in the 1990s as a factor for repressing the clonogenicity, while inducing the differentiation of monocytic leukaemia M1 cells in mice.^26^ LIF functions by interacting with gp130, by which means intracellular signalling is transduced in neurons and oligodendrocytes, resulting in the increased expression of neuro‐survival associated genes.^27^

In this paper, we established an I/R‐induced hepatic injury model in C57BL/6 mice, following established procedures^28^, ^29^ and hypoxia‐induced cell line model in NCTC‐1469 cells. By using a knockdown strategy, histological approaches and molecular methods, we further demonstrated that remifentanil ameliorates I/R‐induced hepatic injury through the regulation of HIF1α and ZEB1/LIF axis. Our work deciphers the molecular mechanism of hepatic I/R injury, which could provide novel therapeutic strategy for the clinical application.

## 2 METHODS

### 2.1 Animal treatment

A total of 108 male C57BL/6 mice (ageing 8‐12 weeks and weighing 22 ± 2 g) were obtained from Experimental Animal Center of Xi'an Jiaotong University. The mice were randomly assigned into the following nine groups (12 mice in each group): normal, I/R, I/R + remifentanil, I/R + negative control (NC) for shRNA (sh‐NC) (for sh‐HIF1α), I/R + remifentanil +sh‐NC (for sh‐HIF1α), I/R + remifentanil +sh‐HIF1α, I/R + sh‐NC (for sh‐ZEB1), I/R + remifentanil +sh‐NC (for sh‐ZEB1) and I/R + remifentanil +sh‐ZEB1. Alternative mice used as back‐ups for each group were not included in the statistical analysis. All mice were housed with a 12 hour/12 hour light‐dark cycle at a temperature of 20‐25°C, and 60% relative humidity. The mice were acclimated to their environment for 4 days before model establishment.

For hepatic I/R model establishment, the mice were anesthetized by intraperitoneal (i.p.) injection of 30 mg/kg of tiletamine/zolazepam solution supplemented with 10 mg/kg of xylazin.^29^, ^30^, ^31^ After a midline laparotomy, the liver hilum was isolated carefully, followed by placement of a micro‐vascular clamp at the first branch of the liver artery and portal vein supplying the left lateral and median lobes of the liver. The circulation of the caudal lobes remained intact to prevent congestion in the intestinal venous. The mice were placed on a heating pad, and saline soaked sterilized gauze was used to cover the peritoneum to prevent dehydration. Then, 90 minutes after hepatic ischaemia, the micro‐vascular clamp was removed to allow the reperfusion, whereupon abdominal wall was closed with 6‐10 nylon sutures. At 6 hours after reperfusion, whole blood was collected by retro‐orbital puncture, and, upon euthanasia, liver samples were harvested for subsequent experiments.^29^, ^32^, ^33^ The sham operation followed same procedure with omission of vascular occlusion. Before ischaemia, the control group was administrated with 0.9% saline i.p. for three times for 1 minute at 5 minutes intervals. For remifentanil treatment, the hepatic I/R mice were injected (i.p.) with remifentanil (30 μg/kg in total; 091110, Yichang Renfu Pharmaceutical Industry) 3 times, and were then injected (i.p.) twice with 1 μL of lentivirus at a concentration of 5 × 10^8^ TU/mL each time.^33^, ^34^, ^35^ Animal use and experimental procedures were carried out in a protocol approved by the Experimental Animal Ethics Committee of Xi'an Jiaotong University. All experimental animals operating procedures were in line with the United States National Institutes of Health (NIH) laboratory animal care and usage guidelines.

### 2.2 Liver function tests

The serum was isolated, and the levels of blood glucose, lipids, aspartate transaminase (AST), and alanine transaminase (ALT) were measure by an automatic chemistry analyser (Beckman).^36^, ^37^

### 2.3 Haematoxylin and eosin (HE) staining

Mouse livers were dissected, fixed, dehydrated, embedded in paraffin and cut into 4 µm sections. The sections were dewaxed with xylene and rehydrated in graded ethanol as follows: xylene and then toluene for 5 minutes each, followed by 100% ethanol for 2 minutes, 95%, 80% and 75% ethanol for 1 minute each, and distilled H_2_O for 2 minutes. Sections were then stained with haematoxylin for 5 minutes, washed with H_2_O, and rinsed with acid alcohol for 30 seconds, followed by immersion in water bath at 50°C for 5 minutes and eosin staining for 2 minutes. Finally, sections were dehydrated by 2 changes of 95% ethanol for 1 minute each, 2 changes of 100% ethanol for 1 minute each, xylene phenol (3:1) for 1 minute, and 2 changes of xylene for 1 minute of each and then sealed slide with mounting medium. Sections were then observed and photographed under an inverted microscope (XSP‐8CA, Shanghai Optical Instrument Factory).

### 2.4 Cell culture

The NCTC‐1469 cell line was purchased from American Type Culture Collection (ATCC) and cultured with Dulbecco's Modified Eagle's Medium (DMEM) supplemented with 10% horse serum, 10% foetal bovine serum (FBS) and 100 μg/mL penicillin/streptomycin in a 37°C in a 5% CO_2_ incubator. Cells were then incubated under hypoxic condition, 37°C, 95% N_2_ and 5% CO_2_ to mimic ischaemia. Reoxygenation was accomplished next by returning cell culture to an incubator containing 5% CO_2_ and 95% O_2_ for 2 hours.^38^, ^39^ Cells were subjected to 9 different treatment conditions as follows: (a) NCTC‐1469, (b) I/R + NCTC‐1469, (c) remifentanil + I/R + NCTC‐1469 (supplemented with 1 μg/mL of remifentanil), (d) I/R + sh‐NC (for sh‐HIF1α), (e) remifentanil + I/R + sh‐NC (for sh‐HIF1α), (f) remifentanil + I/R + sh‐HIF1α, (g) I/R + sh‐NC (for sh‐ZEB1), (h) remifentanil + I/R + sh‐NC (for sh‐ZEB1) and (i) I/R + remifentanil +sh‐ZEB1.

### 2.5 Cell counting kit‐8 (CCK‐8)

Cells were seeded in a 96‐well plate with a density of 2 × 10^3^ cell/well. A well containing only medium was used for normalization. After 24 hours of transfection, 10 μL of CCK8 was added to wells at 0, 24, 48, 72 and 96 hours, followed by incubation at 37°C for 4 hours. A microplate reader (Bio‐Rad) was employed to measure the optical density (OD) at 450 nm. The ratio of OD_experimental_/OD_control_ was calculated to depict the cell growth curve. Experiments were repeated 3 times.

### 2.6 Flow cytometry

Cells in each group were digested with 0.25% trypsin at 2 days after transfection, followed by addition of RPMI‐1640 medium with 10% foetal bovine serum (FBS) to stop the digestion. Cells were centrifuged at 1000 r/min for 5 minutes, and the supernatant was removed. 70% ethanol was used to fix cells to adjust the cell density to 1 × 10^6^ cell/mL. Next, cells were stained with 10 mL of Annexin V‐FITC/PI (556547, Shuojia Biotech Company) at 4°C for 15‐20 minutes. The extent of apoptosis was analysed by flow cytometry (XL model, Conlter, Company) with an excitation of 480 nm and an emission of 530 nm to detect FITC and more than 575 nm to detect PI. The apoptotic rate was presented as the percentage of apoptotic cells.

### 2.7 Dual‐luciferase reporter gene assay

Wild type (wt) or mutant (mut) promoter region of LIF was introduced into the pGL3‐Basic vector (Promega) to make wt‐LIF promoter and mut‐LIF promoter plasmids. HEK‐293T cells were seeded in a 24‐well plate with a density of 3 × 10^4^/well and cotransfected with (a) wt‐LIF promoter + oe‐NC, (b) wt‐LIF promoter + oe‐ZEB1, (c) mut‐LIF promoter + oe‐NC or (d) mut‐LIF promoter + oe‐ZEB1. After 48 hours og transfection, cells were collected, lysed and analysed with a luciferase reporter assay kit (K801‐200; BioVision, Inc), with measurement of luciferase activity by a Glomax20/20 luminometer (Promega). The experiments were repeated in triplicate.

### 2.8 Real‐time quantitative polymerase chain reaction (RT‐qPCR)

The total RNA was extracted with Trizol (15596026, Invitrogen) and reverse‐transcribed into cDNA using reverse transcription kit (RR047A, Takara) using 20 μL reaction system, 37°C, 15 minutes, 85°C, 5 seconds. Then, RT‐qPCR was performed with the TaqMan MicroRNA Assay and TaqMan^®^ Universal PCR Master Mix using the following cycles: 95°C for 2 minutes, followed by 45 cycles at 95°C for 15 seconds and 60°C for 45 seconds. miRNA was reverse‐transcribed by miRNA First Strand cDNA Synthesis, which was subjected to qPCR using a SYBR Premix Ex Taq kit (RR420A, Takara) in a real‐time PCR machine (ABI 7500, ABI). The qPCR system was set up as follows: SYBR Mix 9 μL, forward primer 0.5 μL, reverse primer 0.5 μL, cDNA 2 μL, RNase‐free dH_2_O 8 μL; 95°C for 10 minutes, 95°C for 15 seconds and 60°C for 1 minute, repeated over 40 cycles. Samples were loaded with 3 replicates. Primers were synthesized by Sangon Biotech Co., Ltd. (Table 1). Glyceraldehyde‐3‐phosphate dehydrogenase (GAPDH) was used as internal reference for mRNA and U6 was used for miRNA. Relative expression of target genes was calculated by the 2^−ΔΔCT^ method. ΔΔCT = Ct_experiment_ − Ct_control_, ΔCT = Ct_target_ − Ct_reference_.^40^

**Table 1:** Primer lists for RT‐qPCR

Gene	Sequence
HIF1α	F 5′‐ACTGCCACGGAGAAACCTG‐3′
	R 5′‐AGAAACTGCCTGCACGATGAG‐3′
ZEB1	F 5′‐ACCGCCGTCATTTATCCTGAG‐3′
	R 5′‐CATCTGGTGTTCCGTTTTCATCA‐3′
LIF	F 5′‐GTCAACACAAGCAACAAAGGTC‐3′
	R 5′‐TCCTTAGCGATCTGTTCACCC‐3′
β‐actin	F 5′‐GTGACGTTGACATCCGTAAAGA‐3′
	R 5′‐GCCGGACTCATCGTACTCC‐3′

Abbreviations: F, Forward primer; R, Reverse primer; RT‐qPCR, real‐time quantitative polymerase chain reaction.

### 2.9 Western blot

Total proteins were isolated from each group and quantified using a bicinchoninic acid kit (Thermo). Subsequently, 30 μg of total protein was loaded and separated by sodium dodecyl sulphate‐polyacrylamide gel electrophoresis (SDS‐PAGE) at a constant 80 V for 35 minutes and 120 V for 45 minutes, and then transferred onto a polyvinylidene difluoride (PVDF) membrane (Amersham), followed by 5% skim milk blocking at room temperature for 1 hour. The membrane was incubated with rabbit anti‐HIF1α (1:1000, ab16066, Abcam), rabbit anti‐ZEB1 (1:2000, ab245283, Abcam), rabbit anti‐LIF (1:1000, ab113262, Abcam) and GAPDH (1:1000, ab37168, Abcam) at 4°C overnight. The next day, the PVDF membrane was incubated with horseradish peroxidase (HRP)‐labelled goat anti‐rabbit IgG (1:10 000, ab6721, Abcam) for 1 hour at room temperature. PVDF was then washed 3 times with PBST for 10 minutes each and developed with a chemiluminescence imager (GE). Image Pro Plus 6.0 (Media Cybernetics) was used to analyse the relative expression of proteins. Experiments were repeated 3 times.

### 2.10 shRNA screening

Mice HIF1α and ZEB1 sequences were obtained from GenBank to design shRNA. BLAST was used to screen out non‐specific siRNAs. The 2 shRNA candidates (listed in Table S1) were inserted into the PsiRNA‐neo vector. After validation by restriction enzyme digestion and sequencing, the constructs were designated as sh‐HIF1α‐1, sh‐H1F1α‐2, sh‐ZEB1‐1 and sh‐ZEB1‐2, all of which were transfected into HOSEpiC cells. Western blot analysis was used to measure the expression level of HIF1α and ZEB1, to select the most effective shRNA for the subsequent experiments.

### 2.11 Statistical analysis

The data analysis was performed using SPSS 21.0 (SPSS Inc). Quantitative data were presented as mean ± standard deviation. Data analysis between 2 groups was analysed by unpaired *t* test. Data of different groups were processed by single factor variance analysis with Tukey's post hoc test. *P* < .05 indicated a significant group difference.

## 3 RESULTS

### 3.1 Identification of hepatic I/R injury mouse model

**Figure 1:**
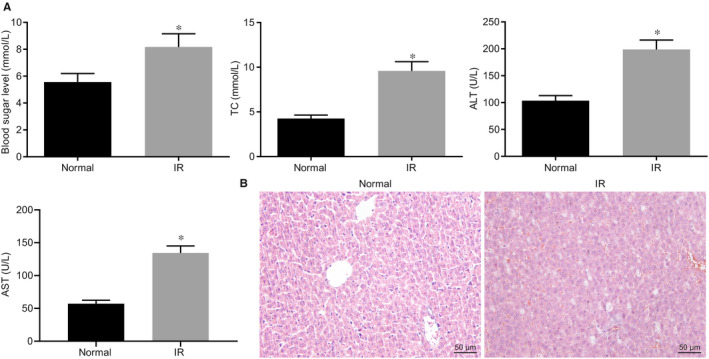
Identification of hepatic I/R injury mouse model. A, Measurements of blood glucose, lipids, AST and ALT levels in the serum. B, HE staining to evaluate the histopathological change in liver tissues (200×, scale bar 50 µm). *P < .05 compared to normal group. Quantitative data are presented as mean ± SD. Data between 2 groups were analysed by unpaired *t* test (12 mice/group)

The serum levels of blood glucose, lipids, AST and ALT were measured after the establishment of the hepatic I/R injury mouse model. The results showed significantly increased levels of all 4 indicators d (*P* < .05) (Figure 1A). The evaluation of histopathology of liver was made after HE staining, which revealed the presence of disorganized hepatic cells, inflammatory cell infiltration, and ballooning degeneration hepatocytes in I/R‐induced hepatic injury (Figure 1B). These data suggested the successful establishment of the hepatic I/R injury mouse model.

### 3.2 Remifentanil can ameliorate hepatic I/R injury

**Figure 2:**
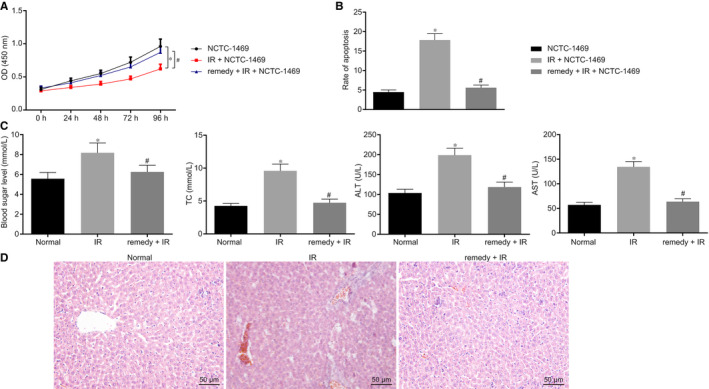
Remifentanil attenuates hepatic I/R injury. A, CCK8 assay for detecting cell proliferation. B, Flow cytometry to analyse cell apoptosis. C, Blood glucose, lipids, AST and ALT evaluation in serum. D, HE staining (200×, scale bar 50 µm). *P < .05 compared to the normal group. ^#^ *P* < .05 compared to I/R + NCTC‐1469. Quantitative data are presented as mean ± SD. Data of different groups were compared by one‐way ANOVA with Tukey's post hoc test (12 mice/group). Experiments were repeated in triplicate

Based on a report that remifentanil can relieve hepatic I/R injury,^34^ we investigated the underlying molecular mechanism by first establishing a hypoxia‐induced I/R model with the NCTC‐1469 cell line. Cells were treated with remifentanil or vehicle, followed by cell viability detection by CCK8 assay (Figure 2A) and cell apoptosis detection by flow cytometry (Figure 2B). The results demonstrated that cell proliferation was notably decreased (*P* < .05), while apoptosis was notably increased in I/R groups compared to the NCTC‐1469 control (*P* < .05). Compared to control, cell proliferation was significantly increased (*P* < .05) while apoptosis was significantly decreased (*P* < .05) in the I/R groups with remifentanil treatment.

Next, we investigated blood markers in the in vivo hepatic I/R injury mouse model with or without remifentanil treatment. Blood glucose, lipids and serum AST and ALT were all significantly elevated in I/R mice compared to the control mice, while compared to I/R groups, the four markers were all notably decreased in I/R mice treated with remifentanil (Figure 2C). HE staining showed that, compared to control mice, hepatic cells in the I/R‐induced hepatic injury model were disorganized, showing ballooning degeneration of hepatocytes and inflammatory cell infiltration. After remifentanil treatment, hepatocytes showed better organization, less inflammatory cell infiltration, and normal morphology compared to I/R groups (Figure 2D). These data indicated that remifentanil could ameliorate hepatic I/R injury.

### 3.3 Remifentanil alleviates hepatic I/R Injury by up‐regulating the expression HIF1α

**Figure 3:**
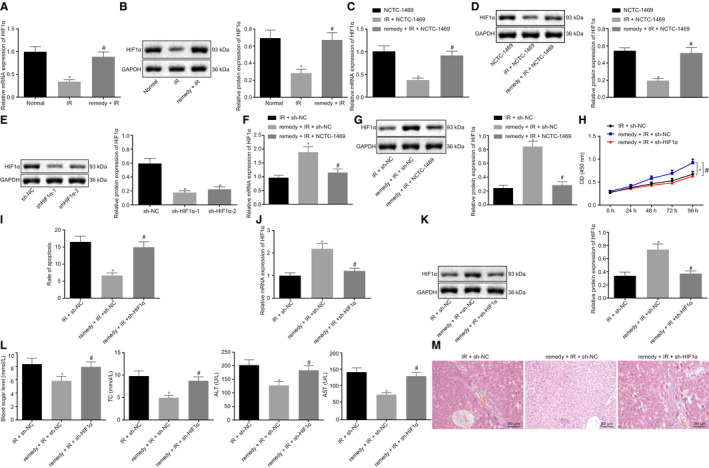
Remifentanil alleviates hepatic I/R Injury by up‐regulating the expression of HIF1α. A, RT‐qPCR to detect the expression of HIF1α in mice liver. B, Western blot to analyse the expression of HIF1α protein. *P < .05 compared to normal group. ^#^ *P* < .05 compared to I/R group. C, RT‐qPCR detection of the expression of HIF1α in the NCTC‐1469 cell line. D, Western blot analysis of the expression of HIF1α protein in NCTC‐1469 cells. *P < .05 compared to NCTC‐1469 cells. ^#^ *P* < .05 compared to I/R + NCTC‐1469 cells. E, Western blot analysis of the expression of HIF1α. *P < .05 compared to sh‐NC cells. F, RT‐qPCR analysis of expression of HIF1α. G, Western blot analysis of the expression of HIF1α protein. H, CCK8 assay of the cell viability. I, Flow cytometry to analyse cell apoptosis. J, RT‐qPCR detection of the expression of HIF1α. K, Western blot analysis of the expression of HIF1α. L, Detection of the level blood glucose, lipids, AST, and ALT. M, HE staining to reveal the histopathological changes in liver (200×, scale bar 50 μm). *P < .05 compared to I/R + sh‐NC cells ^#^ *P* < .05 compared to the remifentanil + I/R + h‐NC group. Quantitative data are presented as mean ± SD. Data of different groups are processed by one‐way ANOVA with Tukey's post hoc test (12 mice/group). Experiments were repeated in triplicate

Since remifentanil reportedly promotes the expression of HIF1α,^16^ we investigated the effects of remifentanil treatment in HIF1α expression in I/R mice model. The results revealed that the expression of HIF1α was remarkably decreased in I/R‐induced hepatic injury mice compared to controls (*P* < .05), while HIF1α expression was remarkably up‐regulated in the mice with remifentanil treatment (*P* < .05) (Figure 3A,B). HIF1α was also measured in cell model, which displayed that, compared to the NCTC‐1469 control cells, HIF1α expression was considerably decreased in hypoxia‐induced NCTC‐1469 (*P* < .05), while HIF1α expression was considerably increased in hypoxia‐induced NCTC‐1469 with remifentanil treatment (*P* < .05) (Figure 3C,D).

To further investigate the role of HIF1α in I/R‐induced hepatic injury, 2 shRNAs were designed to knockdown HIF1α in the NCTC‐1469 cell line. Western blot analysis was used to detect the expression of HIF1α to select the most efficient shRNA for silencing HIF1α. The data demonstrated that HIF1α expression was significantly down‐regulated after sh‐HIF1α‐1 or sh‐HIF1α‐2 treatments compared to sh‐NC (*P* < .05), where sh‐HIF1α‐1 showed the better silencing efficiency, thus justifying its use in subsequent experiments (Figure 3E). In the hypoxia‐induced NCTC‐1469 cell model, RT‐qPCR and Western blot analysis showed that the expression of HIF1α was significantly increased in the remifentanil + I/R + sh‐NC compared to I/R + sh‐NC (*P* < .05). Compared to remifentanil + I/R + sh‐NC, HIF1α expression was significantly lower in the remifentanil + I/R + sh‐HIF1α (*P* < .05) (Figure 3F,G). The CCK8 assay showed that cell proliferation was significantly increased in the remifentanil + I/R + sh‐NC cells compared to I/R + sh‐NC, whereas flow cytometry showed that apoptosis was significantly decreased (both *P* < .05). However, the cell proliferation was significantly decreased while apoptosis was significantly increased in remifentanil + I/R + sh‐H1Fiα compared to remifentanil + I/R + sh‐NC (*P* < .05) (Figure 3H,I).

In I/R‐induced hepatic injury mice model, RT‐qPCR and Western blot were conducted to detect the expression of HIF1α after remifentanil treatment and HIF1α knockdown. The data displayed that, compared to the I/R + sh‐NC group, HIF1α expression was notably up‐regulated in remifentanil + I/R + sh‐NC (*P* < .05). Compared to the remifentanil + I/R + sh‐NC, HIF1α expression was notably down‐regulated in the remifentanil + I/R + sh‐HIF1α cells (*P* < .05) (Figure 3J,K). The evaluation of blood glucose, lipids, AST, and ALT showed that the levels of these indicators were all significantly decreased in remifentanil + I/R + sh‐NC compared to I/R + sh‐NC. However, the levels of these four indicators were all significantly increased in remifentanil + I/R + sh‐HIF1α compared to remifentanil + I/R + sh‐NC (*P* < .05) (Figure 3L). HE staining showed that hepatic cells were well organized and of normal morphology, with less inflammatory cell infiltration in remifentanil + I/R + sh‐NC compared with I/R + sh‐NC. Compared to remifentanil + I/R + sh‐NC treatment, hepatic cells were disorganized, with inflammatory cell infiltration and ballooning degeneration of hepatocytes in remifentanil + I/R + sh‐HIF1α mouse liver. Taken together, these data revealed that remifentanil could relieve hepatic I/R injury by up‐regulating HIF1α expression.

### 3.4 Remifentanil promotes ZEB1 and inhibits LIF expression by up‐regulating HIF1α expression

**Figure 4:**
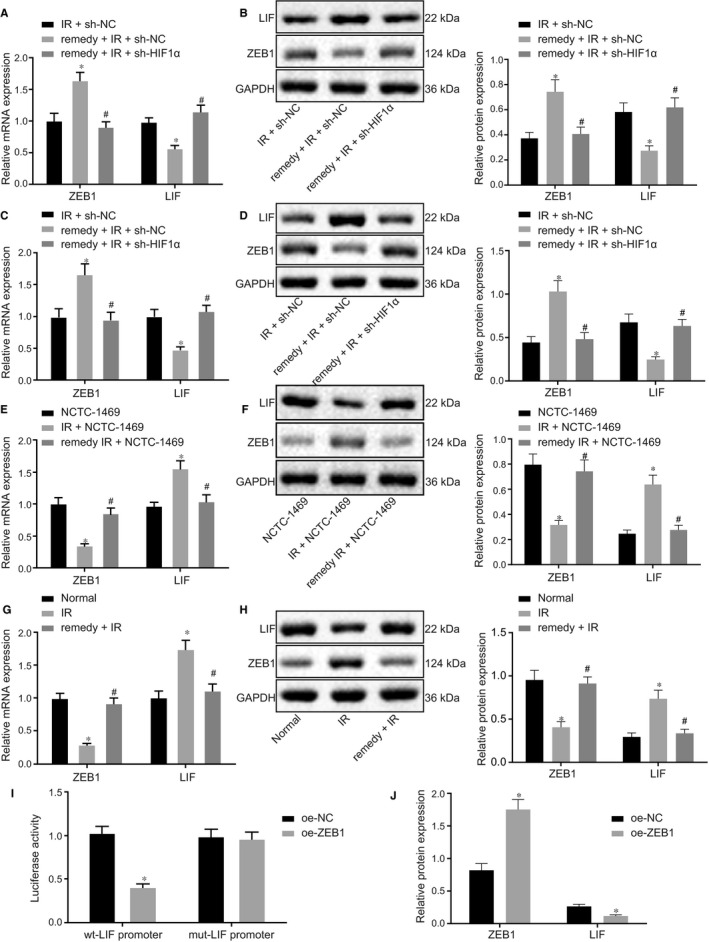
Remifentanil promotes ZEB1 and inhibits LIF expression by up‐regulating HIF1α expression. A, RT‐qPCR detection of the expression of ZEB1 and LIF in cells. B, Western blot detection of the expression of ZEB1 and LIF protein in cells. C, RT‐qPCR detection of the expression of ZEB1 and LIF in mouse liver tissue. D, Western blot detection of the expression of ZEB1 and LIF in mouse liver tissue. *P < .05 in comparison to I/R + sh‐NC; ^#^ *P* < .05 in comparison to remifentanil + I/R + sh‐NC. E, RT‐qPCR assay of the expression of ZEB1 and LIF in cells. F, Western blot assay of the expression of ZEB1 and LIF in cells. *P < .05 in comparison to NCTC‐1469 cells. ^#^ *P* < .05 in comparison to I/R + NCTC‐1469 cells. G, RT‐qPCR analysis to detect the expression of ZEB1 and LIF in mouse liver. H, Western blot analysis of the expression of ZEB1 and LIF protein in mouse liver. *P < .05 in comparison to Normal group. ^#^ *P* < .05 in comparison to the I/R group. I, Dual‐luciferase reporter analysis of the interaction between ZEB1 and LIF. J, Western blot detection of the expression of ZEB1 and LIF proteins in the NCTC‐1469 cell line, *P < .05 in comparison to oe‐NC. Quantitative data are presented as mean ± SD. Data of different groups are processed by one‐way ANOVA with Tukey's post hoc test (12 mice/group). Experiments were repeated in triplicate

Since HIF1α could promote ZEB1 expression,^41^ we treated hypoxia‐induced NCTC‐1469 cell with remifentanil and silenced HIF1α. RT‐qPCR and Western blot analysis showed that ZEB1 expression was considerably increased while LIF expression was considerably reduced in the remifentanil + I/R + sh‐NC group compared to I/R + sh‐NC (*P* < .05). Compared to remifentanil + I/R + sh‐NC treatment, the expression of ZEB1 was significantly lower while the expression of LIF was significantly higher with remifentanil + I/R + sh‐HIF1α treatment (*P* < .05) (Figure 4A,B). RT‐qPCR and Western blot analysis showed that, in comparison to the I/R + sh‐NC group, ZEB1 expression was considerably up‐regulated while LIF expression was considerably down‐regulated in the remifentanil + I/R + sh‐NC group (*P* < .05). However, the expression of ZEB1 was notably down‐regulated, while that of LIF was notably up‐regulated in the remifentanil + I/R + sh‐HIF1α group compared to remifentanil + I/R + sh‐NC group (*P* < .05) (Figure 4C,D). Taken together, these data suggested that remifentanil promotes ZEB1 and inhibits LIF expression by up‐regulating HIF1α expression in I/R‐induced hepatic injury.

Next, RT‐qPCR and Western blot were used detect the expression of ZEB1 and LIF in hypoxia‐induced NCTC‐1469 cell line (Figure 4E,F) and in mouse liver with I/R injury (Figure 4G,H). The results displayed that ZEB1 expression was significantly decreased, while LIF expression was significantly increased in hypoxia‐induced NCTC‐1469 cells and in liver with I/R injury (*P* < .05). After treatment with remifentanil, ZEB1 expression was remarkably increased while LIF expression was remarkably decreased in hypoxia‐induced NCTC‐1469 cells and in liver with I/R injury (*P* < .05). These data indicated that ZEB1 expression was down‐regulated but LIF was up‐regulated in I/R‐induced hepatic injury. Subsequent dual‐luciferase reporter assays revealed that ZEB1 could bind in the promoter region of LIF (Figure 4I). Western blot analysis showed that ZEB1 expression was increased but LIF was decreased in the NCTC‐1469 cell line after overexpression of ZEB1 (*P* < .05) (Figure 4J), indicating that ZEB1 negatively regulated LIF expression and that remifentanil promotes ZEB1 but inhibits LIF expression by up‐regulating HIF1α expression.

### 3.5 Remifentanil ameliorates hepatic I/R injury by regulating ZEB1/LIF axis

**Figure 5:**
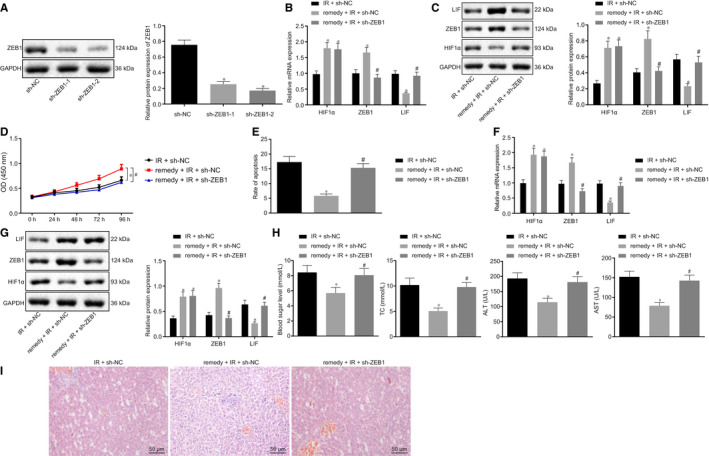
Remifentanil ameliorates hepatic I/R injury by regulating the ZEB1/LIF axis. A, Western blot assay to detect ZEB1 protein expression. *P < .05 in comparison to sh‐NC. B, RT‐qPCR assay of detect the expression of HIF1α, ZEB1, and LIF in the NCTC‐1469 cell line. C, Western blot assay of the expression of HIF1α, ZEB1 and LIF protein. D, CCK8 assay of cell viability. E, Flow cytometry analysis of cell apoptosis. F, RT‐qPCR analysis of the expression of HIF1α, ZEB1 and LIF. G, Western blot analysis of the expression of HIF1α, ZEB1 and LIF protein. H, Detection of the level of blood glucose, lipids, AST and ALT. I, HE staining to show the histopathological changes (200×, scale bar 50 μm). *P < .05 in comparison to I/R + sh‐NC. ^#^ *P* < .05 in comparison to remifentanil + I/R + sh‐NC. Quantitative data are presented as mean ± SD. Data of different groups are compared by one‐way ANOVA with Tukey's post hoc test (12 mice/group). Experiments were repeated in triplicate

Previous data demonstrated that remifentanil could promote ZEB1 and inhibit LIF expression by regulating the expression of HIF1α. To study this in detail, we designed 2 different shRNAs to knockdown ZEB1 in the hypoxia‐induced NCTC‐1469 cell line. Western blot analysis demonstrated that ZEB1 expression in sh‐ZEB1‐1 and sh‐ZEB1‐2 cells was significantly down‐regulated compared to sh‐NC (*P* < .05), where sh‐ZEB1‐2 showed better silencing efficiency, and was thus chosen for the subsequent experiments (Figure 5A). Next, RT‐qPCR and Western blot analysis results displayed that the expression of HIF1α and ZEB1 was notably increased while that of LIF was notably decreased in the remifentanil + I/R + sh‐NC group compared to I/R + sh‐NC group (*P* < .05). Compared to the remifentanil + I/R + sh‐NC group, HIF1α expression was unchanged (*P* > .05) but ZEB1 expression was notably decreased (*P* < .05) and LIF expression was notably increased in the remifentanil + I/R + sh‐ZEB1 group (Figure 5B,C). CCK8 analysis of cell viability (Figure 5D) and flow cytometry for apoptosis (Figure 5E) showed that the cell viability was remarkably increased (*P* < .05) and apoptosis was remarkably decreased (*P* < .05) in the remifentanil + I/R + sh‐NC group compared to I/R + sh‐NC group. Compared to remifentanil + I/R + sh‐NC group, cell viability was remarkably decreased (*P* < .05) and apoptosis was remarkably increased (*P* < .05) in the remifentanil + I/R + sh‐ZEB1 group.

Next, we confirmed the effect of remifentanil on the hepatic I/R injury in mouse models. RT‐qPCR and Western blot analysis showed that, compared to I/R + sh‐NC treatment, HIF1α and ZEB1 expression was considerably increased but LIF expression was considerably decreased in the presence of remifentanil + I/R + sh‐NC treatment (*P* < .05). Compared to remifentanil + I/R + sh‐NC treatment, the expression of HIF1α was unchanged (*P* > .05), while the expression ZEB1 was considerably decreased (*P* < .05) and LIF expression was considerably increased in the remifentanil + I/R + sh‐ ZEB1 group (Figure 5F,G). Analysis of blood glucose, lipids, AST and ALT in serum revealed that the level of these indicators was significantly decreased in remifentanil + I/R + sh‐NC mice compared to the I/R + sh‐NC group (*P* < .05), however compared to remifentanil + I/R + sh‐NC treatment, their level was significantly increased in remifentanil + I/R + sh‐ZEB1 mice (*P* < .05) (Figure 5H). Finally HE staining showed that, in comparison to I/R + sh‐NC treatment, hepatic cells were well arranged and of normal morphology, with fewer inflammatory cells in the remifentanil + I/R + sh‐NC group; in comparison to remifentanil + I/R + sh‐NC, hepatic cells in the remifentanil + IR +sh‐ZEB1 group were disorganized, with abundant inflammatory cell infiltration, along with ballooning degeneration of hepatocytes (Figure 5I). In summary, these data indicated that remifentanil could ameliorate the hepatic I/R injury by regulating the ZEB1/LIF axis.

## 4 DISCUSSION

Hepatic I/R injury after transplantation or surgery is a major reason of liver failure. In recent years, remifentanil has gained increasing attention as a protective agent against I/R‐induced injury in liver, intestine, uterus, heart and other organs.^5^, ^34^, ^42^, ^43^, ^44^, ^45^ However, the underlying mechanism of this effect remained to be elucidated. Our work uncovers the molecular mechanism by which remifentanil ameliorates hepatic I/R injury, through promoting ZEB1 and suppressing LIF expression via up‐regulation of HIF1α expression, using both in vitro and in vivo models. These findings could present novel therapeutic targets for the treatment of I/R‐induced hepatic injury.

Yang et al^15^ reported that pre‐conditioning with remifentanil reduced the extent of hepatic I/R injury in rats. Zhao et al^42^ showed that remifentanil pre‐conditioning protected against hepatic I/R injury in a rat model by activating anti‐apoptotic pathways. Liu et al^34^ also used a rat model to show that remifentanil ameliorated hepatic I/R injury. In this paper, we utilized hypoxia‐induced I/R NCTC‐1469 cell model in vitro and the I/R‐induced hepatic injury in vivo mouse model. Upon treating the cell and mouse models with remifentanil, we found that remifentanil could alleviate the I/R‐induced injury. It is known that ischaemia creates a hypoxic microenvironment, in which HIF1α readily interacts with HIF1β to form a heterodimeric transcriptional complex.^46^, ^47^ Others have reported that HIF1α expression was up‐regulated by remifentanil.^16^ In addition, HIF1α has been investigated to protect mice from hepatic ischaemic damage.^48^ Our data in I/R models demonstrated that HIF1α expression was down‐regulated, but that remifentanil treatment rescued the expression of HIF1α. Silencing of HIF1α in NCTC‐1469 cells decreased cell viability and induced apoptosis. In contrast, remifentanil treatment could rescue from these effects of HIF1α knockdown. In the hepatic I/R injury mouse model, we found that remifentanil treatment decreased the serum level of blood glucose, lipids, ALT and AST compared to control mice, all of which indicated protection of hepatic function. The remifentanil treatment alleviated I/R injury in liver, as evidenced by the normal hepatocyte morphology and sparse inflammatory cell infiltration. These data indicate that remifentanil ameliorate hepatic I/R injury by up‐regulating the expression of HIF1α.

Wellner et al^23^ reported that HIF1α could promote the expression of ZEB1, which is a zinc finger transcription factor that has been implicated to participate in embryonic EMT. Bui et al^22^ was the first to demonstrate the protective role of ZEB1 in cerebrocortical ischaemia. Davis et al^49^ revealed that LIF, an interleukin‐6 family member, could protect neurons from an ischaemic environment via up‐regulating antioxidant enzymes. ZEB1 was also found to have an inhibitory transcriptional function by binding to the promoter region of LIF to suppress its expression in glioma cancer stem cells,^25^ which inspired us to investigate the role of HIF1α and the ZEB1/LIF axis in hepatic I/R injury. Our results demonstrated that LIF expression was negatively correlated with ZEB1, and that HIF1α knockdown decreased the expression of ZEB1, but increased the expression of LIF, whereas remifentanil could rescue these effects in vitro and in vivo. Our data suggest that remifentanil regulates the ZEB1/LIF axis via HIF1α, which led us to investigate if remifentanil indeed attenuates I/R‐induced injury by regulating ZEB1/LIF axis. We found that treatment with remifentanil increased the cell viability but decreased apoptosis in vitro. The in vivo data showed that remifentanil reduced the level of blood glucose, lipids, ALT and AST while ZEB1 knockdown increased the level of these indicators. Finally, HE staining confirmed that remifentanil alleviates hepatic I/R injury through the regulation of ZEB1/LIF axis.

**Figure 6:**
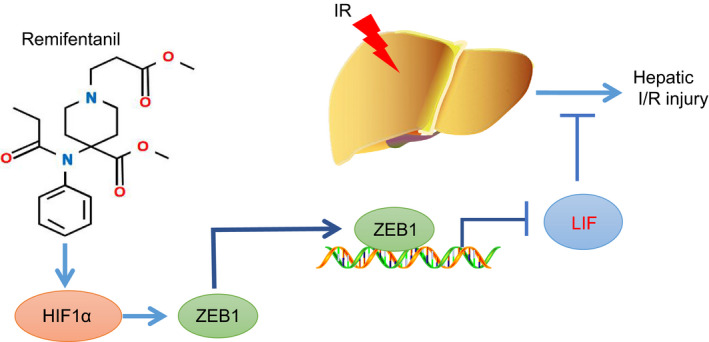
The graphical summary of the function and mechanism of remifentanil to relieve hepatic I/R injury. Remifentanil treatment up‐regulates the expression of HIF1α, which increases ZEB1 expression. ZEB1 can then bind to the promoter region of LIF to inhibit its expression, which ameliorates hepatic I/R injury

In summary, our data demonstrate that remifentanil can ameliorate I/R‐induced hepatic injury by regulating the ZEB1/LIF axis through up‐regulation of HIF1α (Figure 6). For the future clinical applications, our work offers potential therapeutic targets such as by inhibiting LIF expression for the treatment of hepatic I/R injury. However, we note some limitations of our study, which call for further investigations. First, we established an in vitro I/R injury model in NCTC‐1469 cells via hypoxia‐reoxygenation, which may not completely mimic the pathophysiological process of hepatic I/R injury. Besides, the mouse model of hepatic I/R injury was developed according to previous reported studies, including the selection of time of hepatic ischaemia and reperfusion,^29^, ^32^ but there is no accepted standard for these parameters. Therefore, in our future investigations, we shall explore further influence of durations of ischaemia and reperfusion.

## CONFLICT OF INTERESTS

The authors declare that they have no competing interests.

## AUTHOR CONTRIBUTION

**Rongsheng Zhou:** Conceptualization (lead). **Shuang Li:** Investigation (lead); Validation (lead). **Xiaopeng Mei:** Data curation (lead); Visualization (lead). **Tao Jiang:** Writing‐original draft (lead). **Qiang Wang:** Writing‐review & editing (lead).

## Supporting information

Table S1

## Data Availability

Research data not shared.
