# Corrigendum

**DOI:** 10.1111/jcmm.17349

**Published:** 2022-06-06

In Rongsheng Zhou et al.,^1^ incorrect images were used in Figure 1B, Figure 2D, Figure 3M and Figure 5I. The correct figures are shown below. The authors confirm that all results and conclusions of this article remain unchanged.

**FIGURE 1:**
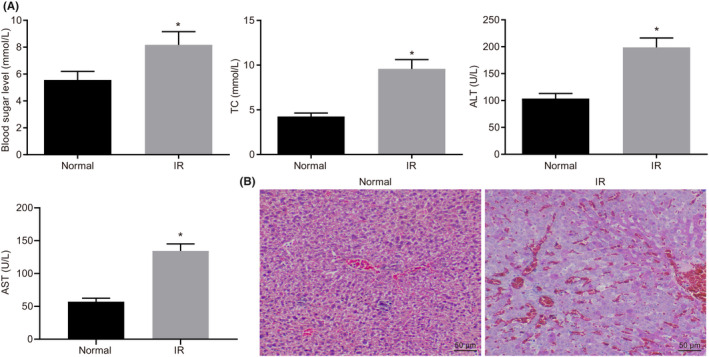
Identification of hepatic I/R injury mouse model. A, Measurements of blood glucose, lipids, AST and ALT levels in the serum. B, HE staining to evaluate the histopathological change in liver tissues (200x, scale bar 50 μm). *p < .05 compared with normal group. Quantitative data are presented as mean ± SD. Data between 2 groups were analysed by unpaired *t* test (12 mice/group)

**FIGURE 2:**
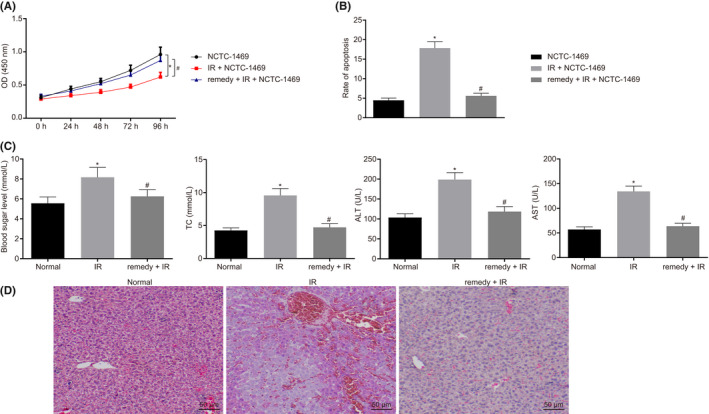
Remifentanil attenuates hepatic I/R injury. A, CCK8 assay for detecting cell proliferation. B, Flow cytometry to analyse cell apoptosis. C, Blood glucose, lipids, AST and ALT evaluation in serum. D, HE staining (200×, scale bar 50 μm). *p < .05 compared with the normal group. #*p *< .05 compared with I/R + NCTC‐1469. Quantitative data are presented as mean ± SD. Data of different groups were compared by one‐way ANOVA with Tukey's post hoc test (12 mice/group). Experiments were repeated in triplicate

**FIGURE 3:**
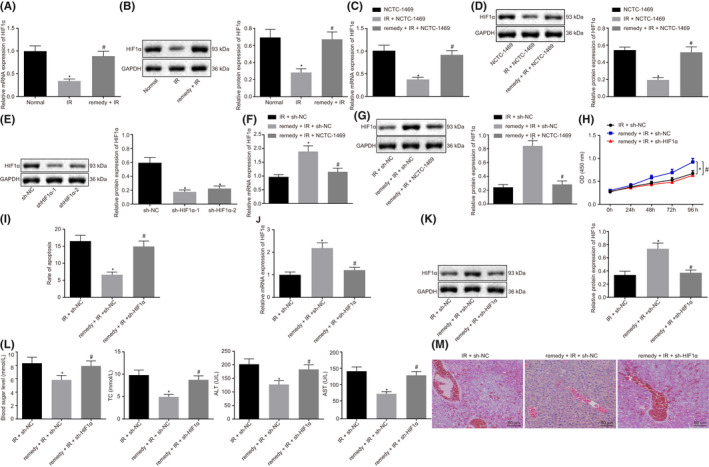
Remifentanil alleviates hepatic I/R Injury by up‐regulating the expression of HIF1α. A, RT‐qPCR to detect the expression of HIF1α in mice liver. B, Western blot to analyse the expression of HIF1α protein. *p < .05 compared with normal group. #*p *< .05 compared with I/R group. C, RT‐qPCR detection of the expression of HIF1α in the NCTC‐1469 cell line. D, Western blot analysis of the expression of HIF1α protein in NCTC‐1469 cells. *p < .05 compared with NCTC‐1469 cells. #*p *< .05 compared with I/R + NCTC‐1469 cells. E, Western blot analysis of the expression of HIF1α. *p < .05 compared with sh‐NC cells. F, RT‐qPCR analysis of expression of HIF1α. G, Western blot analysis of the expression of HIF1α protein. H, CCK8 assay of the cell viability. I, Flow cytometry to analyse cell apoptosis. J, RT‐qPCR detection of the expression of HIF1α. K, Western blot analysis of the expression of HIF1α. L, Detection of the level blood glucose, lipids, AST and ALT. M, HE staining to reveal the histopathological changes in liver (200×, scale bar 50 μm). *p < .05 compared with I/R + sh‐NC cells #*p *< .05 compared to the remifentanil+I/R+ h‐NC group. Quantitative data are presented as mean ± SD. Data of different groups are processed by one‐way ANOVA with Tukey's post hoc test (12 mice/group). Experiments were repeated in triplicate

**FIGURE 5:**
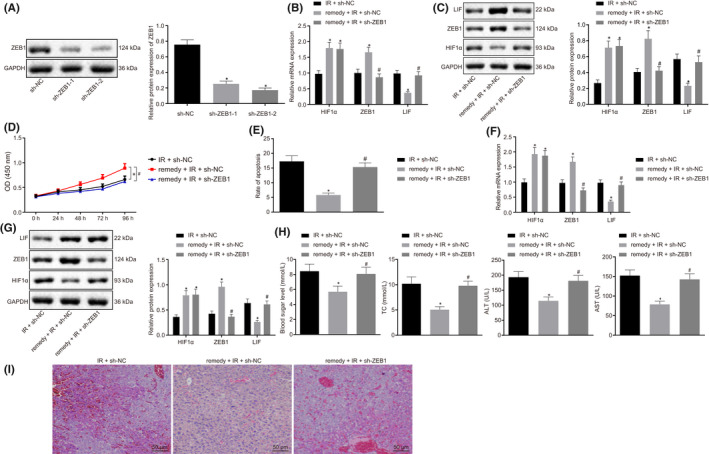
Remifentanil ameliorates hepatic I/R injury by regulating the ZEB1/LIF axis. A, Western blot assay to detect ZEB1 protein expression. *p < .05 in comparison with sh‐NC. B, RT‐qPCR assay of detect the expression of HIF1α, ZEB1 and LIF in the NCTC‐1469 cell line. C, Western blot assay of the expression of HIF1α, ZEB1 and LIF protein. D, CCK8 assay of cell viability. E, Flow cytometry analysis of cell apoptosis. F, RT‐qPCR analysis of the expression of HIF1α, ZEB1 and LIF. G, Western blot analysis of the expression of HIF1α, ZEB1 and LIF protein. H, Detection of the level of blood glucose, lipids, AST and ALT. I, HE staining to show the histopathological changes (200×, scale bar 50 μm). *p < .05 in comparison with I/R + sh‐NC. #*p *< .05 in comparison with remifentanil+I/R+ sh‐NC. Quantitative data are presented as mean ± SD. Data of different groups are compared by one‐way ANOVA with Tukey's post hoc test (12 mice/group). Experiments were repeated in triplicate
