# Supplementary material for: Remifentanil up‐regulates HIF1α expression to ameliorate hepatic ischaemia/reperfusion injury via the ZEB1/LIF axis
Source: J Cell Mol Med. 2020 Sep 30;24(22):13196–207. doi: 10.1111/jcmm.15929 (PMC7701522; doi:10.1111/jcmm.15929)
Supplement: Supplementary file 1 — Table S1 [file JCMM-24-13196-s001.docx]

**Table 1**. Primer lists for RT-qPCR

| Gene | Sequence |
| --- | --- |
| HIF1α | F 5’- ACTGCCACGGAGAAACCTG -3’  R 5’- AGAAACTGCCTGCACGATGAG -3’ |
| ZEB1 | F 5’- ACCGCCGTCATTTATCCTGAG -3’ |
|  | R 5’- CATCTGGTGTTCCGTTTTCATCA -3’ |
| LIF | F 5’- GTCAACACAAGCAACAAAGGTC -3’ |
|  | R 5’- TCCTTAGCGATCTGTTCACCC -3’ |
| β-actin | F 5’- GTGACGTTGACATCCGTAAAGA -3’ |
|  | R 5’- GCCGGACTCATCGTACTCC -3’ |

Note: RT-qPCR, real time quantitative polymerase chain reaction; F: Forward primer; R: Reverse primer

**Supplemental Table 1**. Sequences of shRNA and negative control

| Gene | Sequence (5’-3’) |
| --- | --- |
| sh-HIF1α -1 | GAGAAACCTGCTGCTGCAAAGAAGA |
| sh-HIF1α -2 | GCGGGATACGATGTGGAGAAGAACA |
| sh-ZEB1-1 | AGACCACTGAGGAGGAACCTCTCAA |
| sh-ZEB1-2 | CACTGAGGAGGAACCTCTCAACTTA |
| Negative control | TTCTCCGAACGTGTCACGTTT |
